# Advances in Chinese herbal medicine for diabetic wound treatment: from tradition to innovation

**DOI:** 10.1186/s13020-026-01480-3

**Published:** 2026-08-04

**Authors:** Zhiqun Hong, Xinyi Liu, Bo Liu, Ye Hu, Huaqiong Li, Zhiqiang Yan, Bo Li

**Affiliations:** 1 Department of Orthopedics, The 95th Hospital of Putian, Putian, 351100 Fujian China; 2 Zhejiang Key Laboratory of Soft Matter Biomedical Materials, Wenzhou Institute, University of Chinese Academy of Sciences, Wenzhou, 325000 Zhejiang China; 3 School of Pharmaceutical Science, Zhejiang Chinese Medical University, Hangzhou, 311402 Zhejiang China; 4 Xiangshan Hospital of Wenzhou Medical University, Ningbo, 315700 Zhejiang China; 5 Army Infantry Academy, Nanchang, 330103 Jiangxi China

**Keywords:** Diabetic wounds, Chinese herbal medicine, Hydrogels, Exosomes

## Abstract

Diabetic wounds represent one of the most intractable complications of diabetes, arising from a highly complex and self-perpetuating pathological microenvironment. This milieu is characterized by chronic inflammation, oxidative stress, impaired angiogenesis, peripheral neuropathy, immune dysregulation, recurrent infection, and marked dysfunction of resident cells and the extracellular matrix (ECM). These intertwined abnormalities not only hinder wound closure but also significantly increase the risk of chronic infection, amputation, and disability, thereby imposing substantial clinical and socioeconomic burdens. Conventional therapies, which typically target isolated aspects of wound pathology, often fail to disrupt the vicious cycle of non-healing, underscoring the urgent need for integrated, multi-target therapeutic strategies. Chinese herbal medicine (CHM) encapsulates a holistic therapeutic paradigm that is intrinsically aligned with the multi-factorial nature of diabetic wound management. CHM offers a diverse array of bioactive ingredients, including polysaccharides, saponins, flavonoids, alkaloids, and phenolics, that collectively exert anti-inflammatory, antioxidant, pro-angiogenic, neuroprotective, immunomodulatory, antimicrobial, and pro-proliferative effects. Moreover, many of these ingredients exhibit pleiotropic effects, enabling concomitant modulation of multiple pathological pathways to address the inherent complexity of diabetic wounds. However, the clinical translation of raw CHM is frequently hindered by poor bioavailability and rapid degradation at the wound site. Recent advances in CHM-based innovative delivery platforms have provided a transformative framework to overcome these limitations. This review highlights the emergence of CHM-loaded hydrogels and CHM-derived plant exosome-like nanovesicles (PELNs) as next-generation interventions. CHM-loaded hydrogels act as biomimetic scaffolds that facilitate controlled release and adaptively respond to microenvironmental cues (e.g., pH and reactive oxygen species (ROS)). Simultaneously, CHM-derived exosome-like nanovesicles serve as natural, biocompatible nanocarriers that enhance cellular uptake and molecular signaling, promoting regenerative healing through intercellular communication pathways. In this review, we systematically summarize the pathological features of diabetic wounds and synthesize the modern pharmacological mechanisms of representative CHM ingredients. We further evaluate the synergistic integration of CHM with advanced biomaterials and nanotechnology, emphasizing their translational potential from preclinical models to clinical evidence. By bridging traditional wisdom with cutting-edge bioengineering, these platforms offer a robust evidence-based framework for the modernized management of diabetic wounds.

## Introduction

Diabetes mellitus is a chronic metabolic disorder characterized by persistent hyperglycemia due to impaired insulin secretion or insulin action [1, 2]. The global prevalence of diabetes is increasing rapidly. According to the International Diabetes Federation (IDF), 536.6 million adults (aged 20–79 years) were living with diabetes in 2021, a figure that is projected to reach 783.2 million by 2045 [3]. Among its complications, diabetic wounds—particularly chronic foot ulcers—pose significant threats to patients’ quality of life and survival [3, 4]. Epidemiological data indicate that up to one-third of diabetic patients will develop foot ulcers during their lifetime; among these patients, more than half develop infections, and nearly 20% of infected cases require lower-limb amputation [4]. The 5-year mortality rate following major amputation can exceed 70%, rivaling that of many malignancies [5].

The pathogenesis of diabetic wounds is highly complex. Rather than resulting from a single defect, diabetic wounds arise from a convergence of chronic inflammation, oxidative stress, impaired angiogenesis, peripheral neuropathy, immune dysregulation, recurrent infection, and dysfunction of resident cells and the extracellular matrix (ECM) [6–8]. Hyperglycemia-driven metabolic abnormalities, accumulation of advanced glycation end products (AGEs), and overactivation of inflammatory pathways (e.g., NLR family pyrin domain containing 3 (NLRP3) inflammasome and nuclear factor-κB (NF-κB)) together create a microenvironment characterized by sustained oxidative stress and inflammatory damage [9–11]. This not only impairs the function of endothelial cells, neurons, fibroblasts, and keratinocytes, thereby further impairing angiogenesis and aggravating peripheral neuropathy, but also compromises host defense, leading to persistent M1 macrophage polarization, neutrophil infiltration, and recurrent infection [12–14]. These processes also reinforce one another, resulting in a self-perpetuating pathological cycle that is highly resistant to conventional interventions. Current standard treatments, including wound dressings, debridement, revascularization, and negative pressure wound therapy (NPWT), typically target isolated aspects of wound pathology and are often insufficient to break the vicious cycle of chronic non-healing wounds [15]. As such, the prevalence of chronic, non-healing diabetic wounds remains unacceptably high. There is an urgent clinical need for new therapeutic strategies that can address the multifactorial and dynamic nature of diabetic wound pathophysiology.

Chinese herbal medicine (CHM), with its long history of clinical use and rich empirical foundation, offers a holistic and multi-target approach to wound management [16]. These pleiotropic effects are attributable to a diverse array of bioactive ingredients, including polysaccharides, saponins, flavonoids, alkaloids, and polyphenols, which collectively exert anti-inflammatory, antioxidant, pro-angiogenic, immunomodulatory, and antimicrobial effects [17, 18]. Accumulating clinical and experimental evidence demonstrates that CHM can promote wound closure, enhance granulation tissue formation, regulate immune and inflammatory responses, and reduce the risk of infection [19].

In recent years, advances in biomaterials science and nanotechnology have led to the development of innovative CHM-based delivery platforms, including CHM-loaded hydrogels and CHM-derived plant exosome-like nanovesicles (PELNs) [20, 21]. CHM-loaded hydrogels not only provide a biocompatible, moist environment for wound healing, but also enable controlled and sustained release of herbal bioactives. Meanwhile, PELNs—acting as natural nanocarriers for diverse bioactive molecular cargo, including microRNAs (miRNAs), proteins, and small-molecule ingredients—facilitate transdermal delivery and augment cellular uptake, thereby substantially broadening the therapeutic landscape of CHM [22–24].

To ensure a comprehensive and systematic collection of literature, a thorough search was performed across PubMed, Web of Science**,** Embase, CNKI, and Google Scholar, primarily covering publications from January 2000 to December 2025. The search strategy utilized combinations of keywords including: (“*diabetic wound*” OR “*diabetic foot ulcer*” OR “*diabetic skin injury*”) AND (“*Chinese herbal medicine*” OR “*traditional Chinese medicine*” OR “*TCM*”) AND (“*wound healing*” OR “*tissue repair*” OR “*regeneration*”) AND (“*hydrogel*” OR “*exosome*” OR “*extracellular vesicles*” OR “*exosome-like nanovesicle*”). Following the initial search, we manually screened the retrieved articles and their references, prioritizing original research and high-impact studies that provide significant mechanistic insights or clinical evidence to support the discussions in this review.

In this review, we provide a comprehensive overview of the pathological features of diabetic wounds and systematically summarize the mechanisms by which CHM and its bioactive ingredients modulate key wound-healing pathways. Particular emphasis is placed on recent advances in CHM-loaded hydrogels and PELNs, including their underlying mechanisms, preclinical and early clinical evidence, and translational potential. Through this synthesis, we aim to highlight critical knowledge gaps, inspire future research, and foster the development of novel, evidence-based multi-target therapies for chronic diabetic wounds.

## Pathophysiological hallmarks of diabetic wounds

### Oxidative stress and chronic inflammation as central drivers

Oxidative stress and chronic inflammation are central, mutually reinforcing mechanisms that contribute to the impaired healing of diabetic wounds. Under physiological conditions, wound healing proceeds through four highly coordinated phases—hemostasis, inflammation, proliferation, and remodeling—orchestrated by interactions between resident skin cells and infiltrating immune cells [25, 26]. In diabetes, persistent hyperglycemia profoundly disrupts these processes, creating a pathological environment hostile to repair [27].

Sustained hyperglycemia triggers a cascade of metabolic disturbances, primarily involving the polyol pathway, the hexosamine pathway, protein kinase C (PKC) activation, and AGE formation. Increased flux through the polyol pathway diverts glucose to sorbitol, consuming nicotinamide adenine dinucleotide phosphate (NADPH) and impairing the regeneration of reduced glutathione (GSH), thereby diminishing cellular antioxidant capacity [53, 54]. Enhanced activity of the hexosamine and PKC signaling (e.g., via activation of NADPH oxidase) further exacerbates oxidative stress and promotes excessive ROS production [28, 29]. Concurrently, accumulation of AGEs—via nonenzymatic glycation of proteins and lipids—can damage cellular macromolecules and the ECM and activate the receptor for AGEs (RAGE), thereby amplifying oxidative stress and tissue injury [11].

This persistent redox imbalance activates key inflammatory signaling pathways, particularly NF-κB and the NLRP3 inflammasome. Their activation drives sustained production of pro-inflammatory cytokines such as tumor necrosis factor-α (TNF-α), interleukin-6 (IL-6), and interleukin-1β (IL-1β), maintaining a non-resolving, deleterious inflammatory milieu [10]. Meanwhile, metabolic dysregulation leads to lactate accumulation and stabilization of hypoxia-inducible factor-1α (HIF-1α), which may further promote NF-κB–mediated pro-inflammatory signaling. Hyperglycemia also increases Notch signaling in immune cells, promoting further cytokine release and sustaining a pro-inflammatory state [30].

**Fig. 1:**
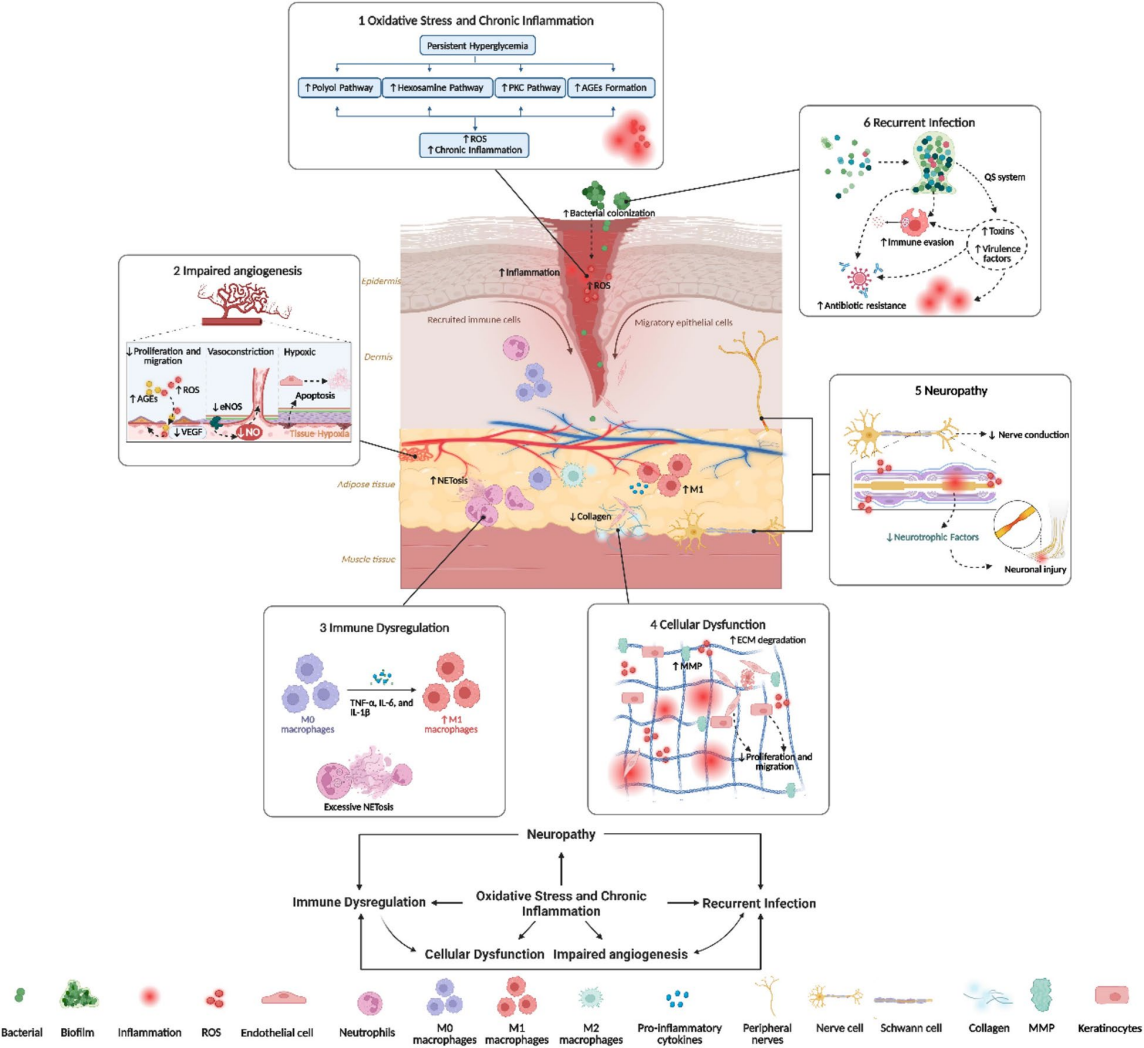
Schematic illustration of pathophysiological characteristics of diabetic wounds. Persistent hyperglycemia disrupts four key metabolic pathways (polyol, hexosamine, PKC, and AGEs formation), collectively leading to excessive ROS production and chronic inflammation. The oxidative stress and chronic inflammation further exacerbate impaired angiogenesis, peripheral neuropathy, immune dysregulation, recurrent infection, and cellular dysfunction

The downstream consequences of sustained oxidative stress and inflammation are far-reaching and multifaceted. Endothelial cells, neurons, fibroblasts, and keratinocytes all suffer impaired viability and function in this hostile microenvironment, resulting in impaired angiogenesis, peripheral neuropathy, and delayed tissue repair [31–33]. Importantly, this oxidative stress-inflammation axis further drives immune dysregulation and recurrent infection, ultimately exacerbating the chronicity and severity of diabetic wounds (Fig. 1) [34].

### Impaired angiogenesis

Impaired angiogenesis is a hallmark of diabetic wound pathology and represents a formidable barrier to effective tissue repair. Angiogenesis—the formation of new blood vessels—is essential for delivering oxygen and nutrients to regenerating tissues. In diabetic wounds, however, this process is substantially impaired by both metabolic and oxidative disturbances. Increasing ROS levels induce lipid peroxidation, protein oxidation, and DNA damage, thereby inhibiting endothelial cell proliferation and migration [34]. Simultaneously, the accumulation of AGEs downregulates vascular endothelial growth factor (VEGF) expression and exacerbates pro-inflammatory signaling, further impairing angiogenesis [35, 36]. Hyperglycemia-associated redox imbalance, including NADPH depletion, compromises endothelial nitric oxide synthase (eNOS) activity and reduces nitric oxide (NO) bioavailability [37]. The resultant reduction in NO bioavailability contributes to vasoconstriction, reduced microvascular perfusion, and persistent tissue hypoxia [38, 39].

Hyperglycemia also upregulates peroxisome proliferator-activated receptor-γ coactivator 1α (PGC-1α) in endothelial cells, which induces Notch signaling and blunts Rac/Akt/eNOS activation, making endothelial cells less responsive to pro-angiogenic cues [40]. Concurrently, downregulation of Krüppel-like factor 4 (KLF4) and key cell-cycle regulators (cyclin D1, cyclin D3) further impairs endothelial proliferation and migration and promotes apoptosis [41]. Beyond intrinsic endothelial dysfunction, diabetes also impairs the mobilization and homing of endothelial progenitor cells (EPCs) to sites of injury, and insufficient pericyte recruitment limits the stabilization and maturation of nascent blood vessels [42, 43]. These abnormalities, in aggregate, result in inadequate neovascularization, sustained tissue hypoxia, and nutrient deprivation at the wound site, delaying wound closure and increasing the risk of ulcer recurrence. Notably, persistent ischemia may precipitate ulcer formation even in the absence of overt trauma [28].

### Peripheral neuropathy

Peripheral neuropathy represents another major barrier to effective diabetic wound healing. In diabetes, metabolic dysregulation exacerbates oxidative stress and inflammation, leading to neuronal injury and mitochondrial dysfunction [29, 44, 45]. The consequent downregulation of neurotrophic factors—including nerve growth factor (NGF), brain-derived neurotrophic factor (BDNF), and glial cell line–derived neurotrophic factor (GDNF)—further impairs nerve maintenance and regeneration [46–48]. In addition, osmotic stress induced by sorbitol accumulation disrupts axonal transport and trophic support [32], while hyperglycemia-induced reductions in Na^+^/K^+^-ATPase activity and intracellular ATP levels slow nerve conduction [49]. Schwann cells under oxidative stress may release extracellular vesicles carrying pro-inflammatory cargo, which can further exacerbate nerve dysfunction and contribute to the progression of diabetic peripheral neuropathy (DPN) [29, 50].

Clinically, diabetic neuropathy primarily manifests as sensory deficits, which delay the detection of minor injuries and increase the risk of repetitive trauma. Beyond sensory loss, neuropathy also disrupts neural-immune regulation, in part through Schwann cell dysfunction [51]. In diabetes, sensory nerve injury and neurodegeneration reduce the release of calcitonin gene-related peptide (CGRP), thereby compromising immune cell recruitment and activation within the wound microenvironment and weakening local host defense and tissue repair [49, 51]. Autonomic dysfunction further impairs cutaneous perfusion and sweat regulation, exacerbating tissue vulnerability [51, 52].

### Immune dysregulation

Immune dysregulation is a defining feature of diabetic wounds, which underlies their persistent, non-resolving inflammatory microenvironment. In physiological wound healing, macrophages and neutrophils play coordinated, stage-dependent roles. Neutrophils act as first responders and are rapidly recruited to the wound site for microbial clearance and debris removal. However, their activation and persistence must be tightly controlled to minimize collateral tissue injury [53]. Pro-inflammatory M1-type macrophages predominate early to clear pathogens and debris, and subsequently transition toward an anti-inflammatory, reparative M2-type to resolve inflammation and promote tissue repair [54].

In diabetes, this finely tuned immune balance is disrupted at multiple levels. The migration of neutrophils, which are recruited via chemotactic signals, is delayed or aberrant [55]. AGEs also dysregulate neutrophil responses, promoting aberrant activation while impairing effective adhesion and migration, thereby leading to pathological accumulation and excessive tissue infiltration in the wound [56]. These dysfunctional neutrophils continuously release ROS and proteolytic enzymes, amplifying local tissue damage [56].

Hyperglycemia also promotes excessive formation of neutrophil extracellular traps (NETs)—web-like structures composed of DNA, histones, and antimicrobial peptides that are normally part of the early innate defense [57]. In diabetes, metabolic reprogramming, including increased glycolysis and fatty-acid oxidation, promotes histone acetylation, peptidylarginine deiminase 4 (PAD4)-mediated histone H3 citrullination, and pannexin-1–dependent ATP release/purinergic signaling, collectively driving NETosis [58–60]. The accumulation of NETs can activate the NLRP3 inflammasome, thereby amplifying IL-1β release and oxidative stress [61]. NETs can also induce fibroblast ferroptosis via the endoplasmic reticulum (ER) stress pathway [62], promote endothelial-to-mesenchymal transition (EndMT), and impair angiogenesis by activating p21-activated kinase 2 (PAK2) and suppressing Hippo- Yes-associated protein (YAP) signaling [63].

Macrophage dysfunction further aggravates this pathological state. The shift from M1 to M2 macrophages is impaired in diabetic wounds, resulting in a persistent M1-dominated inflammatory state [64, 65]. Several upstream factors exacerbate this imbalance. For example, AGEs promote autophagy and IRF8 activation in macrophages, driving M1 polarization [66]; ubiquitin-specific protease 7 (USP7) stabilizes GATA3, further biasing macrophages toward pro-inflammatory M1 phenotype [67]; and long noncoding RNA growth arrest-specific 5 (lncRNA GAS5) amplifies signal transducer and activator of transcription 1 (STAT1) signaling, reinforcing this pro-inflammatory milieu [68]. Chronic activation of NF-κB and the NLRP3 inflammasome, triggered by metabolic and oxidative stress, sustains the excessive production of pro-inflammatory cytokines, further reinforcing M1 dominance and perpetuating tissue inflammation [10, 32]. As a result, M2 macrophage populations and their key anti-inflammatory mediators (interleukin-10 (IL-10), transforming growth factor-β (TGF-β)) are diminished, delaying inflammation resolution and tissue repair [69, 70].

In summary, immune dysregulation in diabetic wounds—characterized by sustained M1 polarization, neutrophil dysfunction, and excessive NET formation—traps the wound in a chronic, non-resolving inflammatory state. This maladaptive immune activation prevents timely progression to the proliferative and remodeling phases, ultimately leading to delayed wound closure or persistent non-healing.

### Recurrent infection

Recurrent infection is both a hallmark and a potent amplifier of diabetic wound chronicity. Diabetic wounds are highly susceptible to persistent and recurrent microbial colonization due to a convergence of local and systemic factors and compromised host defense. Chronic hyperglycemia not only creates a nutrient-rich wound milieu that supports microbial proliferation but also exacerbates oxidative stress and tissue damage, which facilitates biofilm establishment and persistence, thereby providing a protective niche for pathogens [71, 72]. In parallel, diabetes-associated immune dysfunction substantially reduces the efficiency of pathogen clearance [8]. Additional alterations in the wound microenvironment further promote microbial persistence and pathogenicity. Tissue hypoxia, resulting from impaired angiogenesis, creates a low-oxygen niche that favors the colonization and enrichment of anaerobes such as *Finegoldia magna* and *Bacteroides fragilis* in diabetic wounds [73]. Alkaline pH, frequently observed in chronic wounds, can modulate biofilm formation and virulence programs of common pathogens such as *S. aureus* and *P. aeruginosa* [74, 75].

Microbial biofilm formation is a major challenge in the management of recurrent infection. Within these biofilms, bacteria are embedded in a self-produced extracellular polymeric substance matrix that shields them from both host immune cells and antibiotics [8]. Bacterial communities also coordinate their behavior through quorum sensing (QS), which not only enhances group survival but also promotes antibiotic tolerance and immune evasion [76, 77]. In *Staphylococcus aureus*, hyperglycemia may enhance accessory gene regulator (agr)-regulated virulence programs, increasing the production of toxins such as α-toxin that damage host cell membranes and impair immune clearance [78–81].

These microbial communities further remodel the wound milieu through proteases, toxins, and signaling molecules that perpetuate inflammation, degrade the ECM, and hinder re-epithelialization and angiogenesis [82]. Consequently, compromised host defenses together with a biofilm-prone wound microenvironment promote persistent colonization and recurrent infection in diabetic wounds, making them difficult to eradicate and prone to relapse.

### Cellular dysfunction

Dysfunction of resident skin cells, particularly fibroblasts and keratinocytes, represents the final common pathway of impaired wound healing in diabetes. Fibroblasts are pivotal for granulation tissue formation, collagen synthesis, and matrix remodeling. In the diabetic environment, however, they exhibit markedly reduced proliferation, migration, and ECM production; these defects are largely attributable to chronic oxidative stress and persistent inflammation [14, 34, 83]. Accumulated AGEs also suppress key regulatory proteins such as far upstream element binding protein 1 (FUBP1), while elevated TNF-α induces insulin-like growth factor 1 (IGF-1) resistance and decreases CD248 expression, together hindering fibroblast function [84].

Keratinocytes, essential for re-epithelialization, are similarly compromised. Hyperglycemia suppresses the p38/MAPK pathway and autophagy, reducing keratinocyte proliferation and migration [85]. Chronic inflammation, characterized by persistent TNF-α elevation and M1 macrophage infiltration, also disrupts the proteolytic balance in the wound niche by increasing matrix metalloproteinase (MMP) activity and reducing tissue inhibitors of metalloproteinases (TIMPs), leading to excessive ECM degradation and impaired re-epithelialization [14, 86].

Mitochondrial dysfunction and impaired energy metabolism represent shared upstream contributors to fibroblast and keratinocyte dysfunction. Under chronic hyperglycemia, activation of hexosamine and NF-κB signaling can upregulate thrombospondin 2 (TSP2), which in turn impairs mitochondrial oxidative phosphorylation (OXPHOS) and reduces cellular energy production [87–89]. In parallel, suppression of FUBP1-mediated Wnt/β-catenin signaling further limits collagen deposition and cellular motility [88]. These changes illustrate that fibroblast and keratinocyte dysfunction does not occur in isolation but reflects the downstream convergence of persistent metabolic dysregulation, oxidative stress, and inflammatory insults throughout diabetic wound pathogenesis.

In summary, diabetic wounds arise from a highly interconnected network of metabolic, vascular, neural, immune, and cellular dysfunctions. These interlocking mechanisms reinforce one another, creating a self-perpetuating pathological environment. Effective intervention therefore requires comprehensive, multi-target strategies to break these vicious pathological cycles and restore effective healing.

## Applications and mechanisms of Chinese herbal medicine in diabetic wound healing

### Traditional formulations and clinical applications

Chinese herbal medicine (CHM), with its intrinsic multi-component, multi-target properties, is uniquely suited for this challenge—coordinating the regulation of inflammation, oxidative stress, immunity, angiogenesis, neuroprotection, ECM remodeling, and antimicrobial defense [16]. The use of CHM for chronic, non-healing ulcers has been documented for over two millennia [90, 91]. As early as the *Huangdi Neijing* (*The Yellow Emperor’s Inner Canon of Medicine*, ca. 3rd–first century BCE), descriptions of toe lesions termed *tuo yong* suggest an early recognition of chronic ulcerative conditions resembling modern diabetic ulcers. Across subsequent dynasties, a rich pharmacopeia of CHM formulations was developed for ulcerative and necrotic lesions. For instance, *Simiao Yong'an Decoction*, recorded in *Hua Tuo Shen Yi Mi Zhuan* (Han Dynasty, ca. second century CE), combines *Lonicera japonica*, *Scrophularia ningpoensis*, *Angelica sinensis*, and *Glycyrrhiza uralensis* and is traditionally used to “clear heat and toxins”, invigorate blood circulation, and promote wound healing. *Yuquan Pill* and *Zihua Decoction* from *Jingyue Quanshu* (late Ming Dynasty, ca. seventeenth century CE), emphasize supplementing Qi and nourishing Yin, which may help support immune and metabolic functions. *Waike Zhengzhi Quansheng Ji* (Qing Dynasty, ca. eighteenth century CE) introduced a range of external therapies, such as *Yanghe Jiening Plaster*, *Zhizhu Ointment*, and *Badu Shengji Powder*, highlighting the integration of systemic and local interventions.

Recent pharmacological studies have further elucidated the molecular mechanisms underlying these classic and modern CHM formulations. For example, *Sanhuang Xiaoyan Formula (SHXY)* has been found to attenuate inflammation and accelerate wound healing by activating the AMP-activated protein kinase (AMPK)/nuclear factor erythroid 2-related factor 2 (Nrf2) signaling and downregulating HMGB1 expression [92]. *Ruyi Jinhuang Powder (RJP)*, reported to contain curcumin, hesperidin, glycyrrhizic acid, and coptisine, promoted fibroblast proliferation and migration via Wnt/β-catenin activation [93]. *Tangzu Yuyang Ointment (TYO)*, containing berberine, ferulic acid, and ginsenosides, has been shown to inhibit bacterial colonization and inflammation while improving local perfusion [94]. Collectively, both classical and recent discoveries underscore CHM’s distinctive capacity to intervene in chronic wound healing and modulate the pathological microenvironment, bridging empirical tradition with modern molecular science [95].

Modern clinical research is increasingly validating the efficacy of these formulas; for instance, *Tangzu Yuyang Ointment* (*TYO*) significantly improved wound closure rates and inflammatory profiles in a prospective randomized controlled trial (RCT) involving chronic diabetic foot ulcer patients [94]. As summarized in Table 1, while several formulas have entered clinical evaluation, much of the current evidence still stems from small-scale observational studies or retrospective series. Therefore, large-scale, multi-center RCTs remain essential to further standardize outcome metrics and confirm the translational potential of these traditional therapies.

**Table 1:** Summary of representative traditional Chinese medicine formulas for diabetic wound healing

Formula name (Chinese/Pinyin)	Main composition	Key active ingredients	Evidence type	Study model	Major outcomes	References
Badu Shengji Powder 拔毒生肌散	Elephas maximus (Xiangpi), Manis pentadactyla (Chuanshanjia), Dracaena cochinchinensis (Longxuejie), Gekko (Shougong), Panax notoginseng (Sanqi), Boswellia carterii (Ruxiang), Commiphora myrrha (Moyao), Paris polyphylla (Qiyeyizhihua), Acacia catechu (Ercha), Moschus (Shexiang)	Mercuric compounds (calomel), heavy metal oxides (red ochre), resin acids, borneol	Clinical (Comparative Study)	DFU patients (n = 140), multicenter, 28-day intervention	28-day total effective rate:91.67% vs 76.47% (silver sulfadiazine); median granulation formation time:20 d vs.28 d; wound healing rate:77.53% vs 66.01%	[212]
Compound Phellodendron Liquid 复方黄柏液	Phellodendron chinense (Huangbai), Forsythia suspensa, Lonicera japonica, Taraxacum mongolicum, Scolopendra (Wugong)	Berberine, forsythoside, chlorogenic acid	Clinical (Cohort Study)	DFU patients (n = 720), 4-week follow-up	Reduced ulcer area and increased growth factors (VEGF, PDGF); more effective than control solution	[213]
Danggui Buxue Decoction 当归补血汤	Angelica sinensis, Astragalus membranaceus	Ferulic acid, astragaloside IV, calycosin, formononetin, ligustilide	Experimental	STZ-induced diabetic mice; GK rats	Ameliorates lipid metabolism and insulin resistance; regulates AGE-RAGE, PI3K/Akt, MAPK, and NF-κB pathways in diabetic complications	[214]
Gubu Decoction 顾步汤	Scutellaria baicalensis, Dendrobium nobile, Angelica sinensis, Lonicera japonica, Glycyrrhiza glabra, Viola philippica, Chrysanthemum indicum, Panax ginseng, Achyranthes bidentata	Scutellarin, dendrobine, ginsenosides	Experimental	STZ-induced diabetic rats	Downregulates HIF-1α; promotes neovascularization; activates TGF-β1/Smad3; reduces miR-155 and M1 markers (iNOS, COX-2); increases FGF7	[215]
Huangqi Guizhi Wuwu Decoction 黄芪桂枝五物汤	Astragalus membranaceus, Cinnamomum cassia (Guizhi), Paeonia lactiflora (Shaoyao), Zingiber officinale (Shengjiang), Ziziphus jujuba (Dazao)	Astragaloside IV, paeoniflorin, cinnamaldehyde	Clinical (Empirical Application)	DFU patients (clinical syndrome differentiation)	Nourishes Qi and warms meridians; improves blood circulation and peripheral neuropathy; clinically modified for diabetic foot	[216]
Jingwanhong Ointment 京万红软膏	30 kinds of Chinese herbs including Angelica sinensis, Papaver somniferum (Yingsuqiao), Dioscorea opposita, Lonicera japonica, etc	Multiple bioactive compounds	Experimental	STZ + sciatic nerve injury rats	Markedly reduces ulcer size and Wagner grade; almost healed after 21 days; increases PDGF mRNA expression	[217]
Kangfuxin Liquid 康复新液	Periplaneta americana extract (rich in peptides, polyols, sticky sugar amino acids)	Peptides, polyols, SSAA	Clinical (Meta-analysis)	11 RCTs (n = 889 DFU patients)	Total effective rate RR = 1.38 (P < 0.00001); cure rate RR = 1.67 (P = 0.005); shortens healing time (MD = − 5.73 days); improves transcutaneous oxygen pressure	[218]
Moist Exposed Burn Ointment (MEBO) 湿润烧伤膏	Coptis chinensis, Phellodendron chinense, Scutellaria baicalensis, Pheretima (Dilong), Papaver somniferum (Yingsuqiao), etc	Berberine, baicalin, puerarin	Clinical	STZ-induced diabetic rats; clinical DFU patients	Promotes re-epithelialization; activates PI3K-Akt-mTOR pathway; wound healing rate significantly higher than control on days 11–25	[219]
Ruyi Jinhuang Powder 如意金黄散	Curcuma longa, Rheum palmatum, Phellodendron chinense, Atractylodes lancea, Magnolia officinalis, Citrus reticulata, Glycyrrhiza uralensis, Arisaema erubescens, Angelica dahurica, Trichosanthes kirilowii	Curcumin, berberine, luteolin, demethoxycurcumin, ar-turmerone	Clinical	STZ-induced diabetic rats; clinical observation	Clinical efficacy up to 92%; reduces ROS and IL-6 in high-glucose fibroblasts; upregulates CD31/VEGF-A; activates Wnt/β-catenin and PI3K-Akt	[93]
Sanhuang Xiaoyan Formula 三黄消炎方	Rheum palmatum, Scutellaria baicalensis, Crataegus pinnatifida, Phellodendron chinense, Isatis tinctoria, Forsythia suspensa, Polygonum bistorta, Commelina communis, Glycyrrhiza uralensis, Coptis chinensis, Polygonum cuspidatum	Berberine, baicalin, emodin, rhein	Clinical	STZ-induced diabetic rats; clinical cohort	Activates AMPK/Nrf2; downregulates HMGB1; reduces MDRO infection in DFU patients; decreases MMP-2 and increases TIMP-1	[92]
Shengji Yuhong Ointment 生肌玉红膏	Angelica sinensis, Lithospermum erythrorhizon (Zi Cao), Angelica dahurica, Glycyrrhiza uralensis, Croton draconoides (Xue Jie), calomel, beeswax	Glycyrrhizic acid (lead compound), ferulic acid	Experimental	Rat full-thickness wound model	Accelerates wound closure; reduces inflammation; promotes collagen deposition; targets PPIA to modulate CD8+ T cells, neutrophils, and memory B cells	[220]
Simiao Yong'an Decoction 四妙勇安汤	Lonicera japonica, Scrophularia ningpoensis, Angelica sinensis, Glycyrrhiza uralensis	Quercetin, kaempferol	Experimental	db/db mice; high-glucose-stimulated cells	Inhibits AGE-RAGE signaling; downregulates TNF-α, IL-6, IL-1β; promotes macrophage polarization	[215]
Tangzu Yuyang Ointment 唐祖玉阳膏	Compound TCM ointment (berberine, ferulic acid, ginsenosides identified)	Berberine, ferulic acid, ginsenosides	Clinical (multicenter RCT)	DFU patients (Wagner 1–2, n = 60), multicenter, 12-week follow-up	Ulcer improvement rate 79.2% vs. 41.7% at 12 weeks (P=0.017); 91.7% vs. 62.5% at 24 weeks (P=0.036); safe with few side effects	[94]
Yanghe Jiening Plaster 阳和解凝膏	Arctium lappa (Niubang), Impatiens balsamina (Fengxian), Aconitum carmichaelii (Fuzi), Cinnamomum cassia (Rougui), Angelica sinensis, Chuanxiong, Paeonia lactiflora (Chishao), Angelica dahurica, Bletilla striata, Ophiopogon japonicus, etc	Cinnamaldehyde, ferulic acid, paeoniflorin	Clinical (Empirical Application)	Chronic non-healing ulcer patients (Yin-type sores)	Warms meridians and disperses cold; promotes blood circulation	[221]
Yuquan Pill 玉泉丸	Pueraria montana, Trichosanthes kirilowii, Ophiopogon japonicus, Rehmannia glutinosa, Schisandra chinensis, Glycyrrhiza glabra	Puerarin, ophiopogonin, schisandrin	Clinical (systematic review)	T2DM patients with complications (systematic review of clinical studies)	Improves insulin sensitivity, glucose/lipid metabolism; protects endothelial cells; reduces VEGF levels in T2DM complications	[222]

### Key bioactive ingredients and their roles

CHM contains a wide array of bioactive ingredients, including polysaccharides, flavonoids, saponins, alkaloids, and phenolic ingredients. These ingredients exhibit diverse biological activities—such as anti-inflammatory, antioxidant, immunomodulatory, pro-angiogenic, neuroprotective, ECM-remodeling, and antimicrobial effects—that collectively target the complex pathophysiology of diabetic wounds (Table 2). Notably, many CHM ingredients display pleiotropic actions, enabling comprehensive therapeutic outcomes.

**Table 2:** Representative CHM-derived bioactive ingredients with pleiotropic activities in diabetic wound healing

Ingredient	Botanical source	Major bioactivities	Main pathways/targets	Reference
Achyranthes bidentata polypeptides	Achyranthes bidentata Blume (root)	Neuroprotective	Schwann cell apoptosis↓ (Nrf2/ARE pathway)	[135–137]
Astragaloside IV	Astragalus membranaceus (Fisch.) Bunge (root)	Immunomodulatory ECM Remodeling Pro-proliferative	M1→M2 polarization (arginase-1, Ym1, IL-13)↑ ECM genes↑ (fibronectin, collagen IIIa)	[155–157]
Astragalus polysaccharide	Astragalus membranaceus (Fisch.) Bunge (root)	Immunomodulatory	M1→M2(β-catenin/NF-κB axis↓)	[142]
Asiaticoside	Centella asiatica (L.) Urb. (leaf)	Anti-inflammatory Antioxidant ECM Remodeling Pro-proliferative	ROS↓, antioxidant enzymes (SOD, NO, GSH-Px, Bcl-2)↑ Wnt/β-catenin pathway↑ Fibroblast proliferation & migration↑	[114, 160]
Asperosaponin VI	Dipsacus asper Wall. ex C.B.Clarke (root)	Pro-angiogenic Circulation-enhancing	HIF-1α/VEGF axis↑ Endothelial cell proliferation↑	[120]
Baicalin	Scutellaria baicalensis Georgi (root)	Anti-inflammatory Antioxidant Pro-angiogenic Circulation-enhancing	Nrf2↑, ROS↓ Ang-1/VEGF-c/TGF-β/Tie-2↑ Re-epithelialization↑	[92, 103]
Berberine	Coptis chinensis Franch.; Phellodendron amurense Rupr. (rhizome/bark)	Anti-inflammatory Antioxidant Immunomodulatory Anti-biofilm ECM Remodeling	IL-17/Cxcl1/MMP9↓ MRSA biofilm↓ (tarO gene↓) MMP9↓, TGF-β1↑, TIMP1↑	[153, 154]
Bletilla polysaccharide	Bletilla striata (Thunb.) Rchb.f. (tuber)	Anti-inflammatory Antioxidant Pro-angiogenic	SOD/GSH↑, MDA↓ NLRP3 inflammasome↓ VEGF↑	[104–107]
Catalpol	Rehmannia glutinosa Libosch. ex Fisch. & Mey. (root tuber)	Neuroprotective	NGF/BDNF expression↑ Neuronal apoptosis↓	[46–48]
Curcumin	Curcuma longa L. (rhizome)	Anti-inflammatory Antioxidant Pro-angiogenic Antimicrobial	NF-κB↓, IL-6/TNF-α↓ VEGF/HIF-1α/SDF-1α/HO-1↑ Disrupt bacterial biofilm	[93, 97, 98]
Ganoderma polysaccharide	Ganoderma lucidum (Curtis) P. Karst. (fruiting body)	Anti-inflammatory Antioxidant Immunomodulatory	Mitochondrial ROS↓ p38 MAPK↓, M2 polarization↑	[108, 141]
Ginsenosides Rb1/Rb2	Panax ginseng C.A.Mey. (root)	ECM Remodeling Pro-proliferative	Collagen synthesis (Rb1)↑, TIMP-1↑ Epidermal regeneration (Rb2) EGF/EGFR↑	[197]
Glycyrrhizin	Glycyrrhiza uralensis Fisch. ex DC. (root/rhizome)	Anti-inflammatory Antioxidant	AMPK/Nrf2 pathway↑ HMGB1 expression↓	[92]
Hesperetin	Citrus reticulata Blanco (fruit peel)	Pro-angiogenic Circulation-enhancing (SIRT3 activation)	Endothelial cell ferroptosis↓	[123, 124]
Loganin	Cornus officinalis Siebold & Zucc. (fruit)	Neuroprotective	Schwann cell pyroptosis↓ ROS & NLRP3↓	[135–137]
Loureirin A/B	Dracaena cochinchinensis (Lour.) S.C.Chen (resin)	Anti-biofilm	Disrupt bacterial membrane Cytoplasmic leakage↑ Oxidative damage↓	[149, 150]
Notoginsenosides (PNS)	Panax notoginseng (Burkill) F.H.Chen ex C.Y.Wu & K.M.Feng (root)	Anti-inflammatory Antioxidant Pro-angiogenic ECM Remodeling	miR-146a-5p/NF-κB↓ VEGF↑ (GSK-3β/β-catenin) Collagen deposition↑	[109, 110]
Paeoniflorin	Paeonia lactiflora Pall. (root)	Neuroprotective Immunomodulatory	Schwann cell apoptosis↓ (Nrf2/ARE) Macrophage polarization (STAT1↓, STAT6↑)	[132, 144, 145]
Phellopterin	Angelica dahurica (Fisch. ex Hoffm.) Benth. & Hook.f. ex Franch. & Sav. (root)	Neuroprotective Pro-angiogenic	Schwann cell migration↑ Axon regeneration↑ HIF-1α/PDGF-β ↑	[133]
Polydatin	Reynoutria japonica Houtt. (root/rhizome)	Neuroprotective	Oxidative damage↓ Mitochondrial biogenesis↑ (SIRT1)	[135–137]
Puerarin	Pueraria montana (Lour.) Merr. (root)	Immunomodulatory ECM Remodeling	M2 polarization via NF-κB/MAPK↓ Collagen synthesis↑ (TGF-β/Smad pathway)	[139, 140, 143]
Quercetin	Gynura divaricata (L.) DC. (leaf)	Anti-inflammatory Antioxidant Neuroprotective Immunomodulatory	AGE-RAGE signaling↓ TLR4/MyD88/IRF-5↓ M2 polarization↑ GAP-43-positive nerve fibers↓	[111–113, 134]
Rehmanniae Radix	Rehmannia glutinosa Libosch. ex Fisch. & Mey. (root tuber)	Pro-angiogenic Circulation-enhancing	VEGF, PDGF, FGF↑ Angiogenesis↑	[130, 131]
Resveratrol	Reynoutria japonica Houtt. (root/rhizome)	Anti-biofilm	Disrupt bacterial membrane SIRT1 activation↑	[149, 150]
Salidroside	Rhodiola rosea L. (root/rhizome)	ECM Remodeling Pro-proliferative	FGF2/HGF/HO-1↑ Fibroblast-to-myofibroblast differentiation↑	[161, 162]
Salvianolic acid B	Salvia miltiorrhiza Bunge (root/rhizome)	Pro-angiogenic Circulation-enhancing	VEGF↑, PECAM-1↑ Angiogenesis & collagen deposition↑	[121, 122]
Sangzhi alkaloids	Morus alba L. (twig)	Anti-inflammatory Antioxidant Pro-angiogenic	NRF2/HO-1/eNOS pathway↑ eNOS/NO↑ Endothelial function improvement	[115, 116]
Triterpenoids from Poria cocos	Poria cocos (Schw.) Wolf (sclerotium)	Pro-angiogenic Immunomodulatory	PI3K/AKT pathway↑ CD31, VEGF↑ M2 polarization↑	[125–127]

#### Anti-inflammatory and antioxidant ingredients

Chronic inflammation and oxidative stress are central obstacles in diabetic wound healing [10, 32, 96]. Importantly, the anti-inflammatory and antioxidant effects of numerous CHM ingredients have been directly validated in diabetic wound models. For instance, Curcumin (*Curcuma longa*) accelerates cutaneous wound healing in streptozotocin (STZ)-induced diabetic rats [97, 98]. Berberine (*Coptis chinensis Franch, Phellodendron amurense*) promotes wound closure in high-fat diet/STZ-induced diabetic rats [67, 99–102]. Baicalin and baicalein (*Scutellaria baicalensis*) [92, 103]—effectively inhibit the NF-κB signaling, suppress the production of pro-inflammatory cytokines (e.g., TNF-α, IL-1β, interleukin-17 (IL-17), IL-6, CC-chemokine ligand 2 (Ccl2), matrix metalloproteinases (MMPs)), and enhance antioxidant enzyme activities (e.g., total antioxidant capacity (T-AOC), superoxide dismutase (SOD), GSH, catalase). CHM Polysaccharides, such as Bletilla striata polysaccharide (*Bletilla striata*) and Ganoderma lucidum polysaccharides (GL-PS, *Ganoderma lucidum*), directly inhibit NLRP3 inflammasome formation and reinforce antioxidant defenses by activating the Nrf2/heme oxygenase-1 (HO-1) pathway in diabetic wound healing [104–107]. GL-PS also prevents MnSOD (Manganese Superoxide Dismutase) nitration and increases glutathione peroxidase (GPx) activities, further enhancing tissue antioxidant capacity [108].

Additionally, some CHM ingredients further modulate specific molecular pathways in diabetic wound healing. Panax notoginseng saponins (PNS, *Panax notoginseng*) exert anti-inflammatory, hemostatic, and analgesic effects by upregulating miR-146a-5p, which enhances binding to IRAK1/TRAF6 (Interleukin-1 Receptor-Associated Kinase 1/TNF Receptor-Associated Factor 6) and inhibits NF-κB activation [109, 110]. Quercetin (*Gynura divaricata*) reduces AGE-RAGE signaling, downregulates inflammatory cytokines (e.g., IL-6, TNF-α), and enhances insulin sensitivity via AKT1 (RAC-alpha serine/threonine-protein kinase) activation in diabetic rat models [111–113]. Other bioactive ingredients—including asiaticoside (*Centella asiatica*) and Polyhydroxy Sangzhi alkaloid (SZ-A, *Ramulus Mori*)—help protect endothelial cells and promote tissue regeneration by reducing oxidative damage and apoptosis. Asiaticoside has been shown to alleviate oxidative injury and suppress apoptosis in high-glucose-challenged Human Umbilical Vein Endothelial Cells (HUVECs) by downregulating p53, Bcl-2 Associated X Protein (Bax), and caspase-3, while upregulating antioxidant defenses (e.g., SOD, NO, GSH-Px, Bcl-2) [114]. SZ-A has been found to exert a potent antioxidant effect on endothelial cells via activation of the NRF2/HO-1/eNOS pathway, thereby promoting vascular repair and wound healing in diabetes [115, 116]. Together, these ingredients mitigate inflammation and oxidative injury through distinct mechanisms, creating a microenvironment conducive to effective tissue repair.

#### Pro-angiogenic and circulation-enhancing ingredients

Impaired angiogenesis and microvascular dysfunction critically limit nutrient delivery and waste removal in diabetic wounds. A diverse array of CHM ingredients has been shown to promote neovascularization and enhance tissue perfusion specifically during diabetic wound healing. Several CHM ingredients, including curcumin, baicalin, PNS, Asperosaponin VI (ASA VI), and SZ-A, have been shown to upregulate pro-angiogenic factors and promote endothelial proliferation and vascular remodeling. Specifically, curcumin increases the expression of VEGF, TGF-β, HIF-1α, SDF-1α (Stromal Cell-Derived Factor 1 alpha), and HO-1 [117]; baicalin upregulates angiopoietin-1 (Ang-1), VEGF-C, TGF-β, and tyrosine kinase receptor 2 (Tie-2) [118]; PNS modulates the GSK-3 β/β-catenin/VEGF pathway, enhancing endothelial proliferation, migration, and nitric oxide (NO) release under high-glucose conditions [119]; ASA VI activates the HIF-1α/VEGF axis [120]; and SZ-A stimulates eNOS production, improving vasodilation [116]. Additionally, salvianolic acid B (SAB, *Salvia miltiorrhiza*), a water-soluble phenolic acid, boosts VEGF and platelet/endothelial cell adhesion molecule (PECAM-1) expression, promoting collagen deposition and angiogenesis [121, 122]. Hesperetin (a flavonoid isolated from *Citri Reticulatae Pericarpium*) protects endothelial cells from ferroptosis via sirtuin 3 (SIRT3) activation, thereby enhancing angiogenesis in a diabetic rat wound model [123, 124].

Beyond individual active ingredients, some CHM extracts and fractions have also shown pro-angiogenic and circulation-enhancing activity. For example, triterpenoid extract (PTE, *Poria cocos*) activates the PI3K/AKT pathway and upregulates CD31 and VEGF, thereby promoting neovascularization and tissue perfusion in diabetic ulcer models [125–127]. *Angelica dahurica* extract stimulates both PI3K/AKT and HIF-1α pathways in endothelial cells, promoting vascularization and wound healing [128, 129]. *Rehmanniae Radix* (RR), containing polysaccharides, isoflavones, and saponins, upregulates VEGF, PDGF, and fibroblast growth factor (FGF) expression, leading to enhanced angiogenesis and microvascular regeneration [130, 131]. Collectively, these single CHM ingredients and complex extracts converge on angiogenic targets, restore angiogenic signaling, reinforce neovascularization and blood circulation, and ultimately accelerate wound healing in diabetes.

#### Neuroprotective/neuroregenerative ingredients

Diabetic wounds are frequently complicated by peripheral neuropathy, which not only impairs sensory function but also disrupts tissue regeneration. Several CHM ingredients have been shown to exhibit neuroprotective and neuroregenerative effects directly relevant to diabetic wound repair. For instance, paeoniflorin activates the Nrf2/antioxidant pathway and inhibits Schwann cell apoptosis by increasing Bcl-2 and reducing Bax and Caspase-3 expression, thereby preserving neural cell viability under high-glucose conditions [132]. Other ingredients, such as phellopterin (PP, *Angelica dahurica*), enhance Schwann cell migration, axon regeneration, and myelin reconstruction by upregulating axon regeneration marker Tuj1 and myelin basic protein (MBP) in diabetic neuropathy models [133]. Quercetin facilitates the formation of growth-associated protein 43 (GAP-43)-positive nerve fibers, which accelerates neuroregeneration [134]. Ingredients such as polydatin, loganin, and *Achyranthes bidentata* polypeptides have shown neuroprotective effects in preclinical diabetic models [135–137]. Extracts of *Bletilla striata* also mitigate oxidative stress and suppress MAPK-mediated apoptosis, further protecting neural cells from injury [138].

#### Immunomodulatory ingredients

Impaired immune regulation, characterized by dysfunctional macrophage polarization, abnormal neutrophil infiltration, and excessive NETs formation, is another hallmark of diabetic wounds [64, 65, 69, 70]. Persistent activation of pro-inflammatory M1 macrophages and excessive NETs release sustain chronic inflammation, impairing tissue repair. Recent studies have shown that CHM ingredients can restore immune balance by suppressing pro-inflammatory signaling, promoting M2 macrophage polarization, and modulating neutrophil responses.

For instance, puerarin (*Pueraria montana*), an isoflavone flavonoid, suppresses inflammatory cytokine production and promotes M2 polarization by inhibiting NF-κB/MAPK pathways in the treatment of Type 2 Diabetes mouse model [139, 140]. Ganoderma lucidum polysaccharide (GLP, a polysaccharide) enhances M2 polarization by suppressing p38 MAPK signaling [141]. Both astragalus polysaccharide (APS) and astragaloside IV (AS-IV; extracted from *Astragalus membranaceus*) facilitate the M1-to-M2 phenotypic shift: APS acts via inhibition of NF-κB and GSK-3β signaling [142], while AS-IV upregulates the expression of arginase-1, Ym1 (Chitinase-like protein 3), and IL-13, key markers of M2 polarization [143]. Paeoniflorin (PF, extracted from *Paeonia lactiflora Pall.*), a monoterpene glycoside, modulates macrophage phenotype by downregulating STAT1 and activating signal transducer and activator of transcription 6 (STAT6), thus favoring M2 polarization in diabetic wounds [144, 145]. Quercetin (a flavonoid) inhibits the toll-like receptor 4 (TLR4)/myeloid differentiation primary response 88 (MyD88) axis as well as NF-κB and interferon regulatory factor 5 (IRF-5), further promoting a reparative immune phenotype in diabetic rats [146, 147]. Recent research also suggests that certain polysaccharides and flavonoids regulate neutrophil recruitment and inhibit excessive NETs formation, further contributing to the resolution of chronic inflammation and the restoration of immune homeostasis in diabetic wounds [148].

#### Antimicrobial and anti-biofilm ingredients

Recurrent infection and biofilm formation pose significant challenges in diabetic wound healing. Recent studies show that various CHM ingredients exhibit broad-spectrum antimicrobial and anti-biofilm activities, targeting multiple stages of the bacterial lifecycle and biofilm formation.

Phenolic compounds and polyphenols, including resveratrol and loureirin A/B (*Dracaena cochinchinensis*), disrupt bacterial cell membranes, induce cytoplasmic leakage and cause oxidative damage, promoting wound healing in diabetic mice and attenuating the expression of pro-inflammatory cytokines in wound tissue [149, 150]. Curcumin, in addition to its well-documented anti-inflammatory role, exhibits broad-spectrum antibacterial activity by inhibiting bacterial DNA replication, virulence factors expression, and biofilm formation in diabetic wound contexts [151, 152]. Berberine (BBR), a protoberberine alkaloid, inhibits biofilm formation and virulence factor expression by downregulating *tarO* gene expression and compromising bacterial cell wall integrity [153, 154]. These effects have been confirmed in diabetic wound models, highlighting BBR’s therapeutic potential in managing recurrent infections. Other CHM ingredients—such as baicalin, quercetin, and astragaloside IV, also inhibit bacterial growth and biofilm formation by modulating oxidative stress, altering membrane permeability, and disrupting bacterial communication pathways in the diabetic wound milieu [155–157].

#### Proliferation-promoting and ECM remodeling ingredients

Impaired cell proliferation and abnormal ECM metabolism are major barriers to tissue regeneration in diabetic wound healing. Multiple CHM ingredients have demonstrated the ability to promote cellular proliferation and ECM remodeling under diabetic conditions. Ginsenoside Rb1 and Rb2 (*Panax ginseng*) enhance the proliferation of diabetic fibroblasts and keratinocyte in vitro. Specifically, ginsenoside Rb1 upregulates tissue inhibitors of metalloproteinases-1 (TIMP-1) and collagen synthesis in diabetic wound models [158], while Rb2 promotes epidermal regeneration [159]. Asiaticoside facilitates fibroblast proliferation and migration under hyperglycemic conditions by activating the Wnt/β-catenin pathway, supporting granulation tissue formation and re-epithelialization [160].

Salidroside (*Rhodiola rosea*), a phenylethanoid glycoside, induces the expression of HO-1, fibroblast growth factor 2 (FGF2), and hepatocyte growth factor (HGF), promoting fibroblast-to-myofibroblast differentiation, collagen contraction, and ECM remodeling in a diabetic rat model [161, 162]. Puerarin also stimulates the TGF-β/Smad pathway, leading to increased collagen synthesis and matrix organization [163]. AS-IV upregulates ECM-related gene expression, such as fibronectin and collagen IIIa, thereby increasing collagen deposition in diabetic wound tissue [143]. BBR contributes to ECM homeostasis by downregulating matrix metalloproteinase 9 (MMP9) and upregulating transforming growth factor-β1 (TGF-β1) and TIMP1, improving ECM synthesis and tissue repair in diabetic wound contexts [164].

In conclusion, pharmacological studies confirm the multi-target benefits of CHM ingredients in diabetic wound healing. However, clinical application of CHM is limited by the complexity of herbal ingredients, lack of high-quality clinical evidence, and insufficient standardization. The bioavailability of active ingredients is also limited with conventional delivery methods. These challenges have stimulated the development of innovative CHM-based delivery platforms, which are discussed in the following sections.

### Limitations of conventional CHM preparations in diabetic wound management

Despite the theoretical potential of CHM, the clinical translation of raw herbal preparations is frequently hindered by the inherent limitations of traditional dosage forms. Conventional topical formulations—such as herbal decoctions, powders, and hard plasters—suffer from uncontrolled release kinetics, poor swelling capacity, inadequate air permeability, and an inability to maintain the optimal moist healing environment required for chronic wounds [165–167]. Specifically, the burst-release profile of topical decoctions fails to sustain stage-matched therapeutic concentrations required across the phased healing cascade (inflammation, proliferation, and remodeling). Moreover, in the protease-rich, hyperglycemic microenvironment of diabetic ulcers, free bioactive ingredients undergo rapid enzymatic degradation and exhibit poor trans-eschar bioavailability. Furthermore, their tendency to adhere to the highly exuding wound surface necessitates frequent dressing changes, which often causes secondary mechanical trauma and disrupts the fragile, newly forming neoepithelium [168]. Recent advances in biomaterials have provided a transformative framework to overcome these barriers, positioning CHM-loaded hydrogels and CHM-derived exosome-like nanovesicles (PELNs) as next-generation interventions [20, 21].

## Innovations in CHM-loaded hydrogels

Traditional CHM dressings for diabetic wounds are limited by uncontrolled drug release, inadequate air permeability, poor swelling capacity, and insufficient barrier function [165–167]. Their tendency to adhere to the wound surface increases the risk of secondary injury during dressing changes [168]. These limitations underscore the need for advanced delivery platforms that optimize the therapeutic potential of CHM while minimizing adverse effects.

**Fig. 2:**
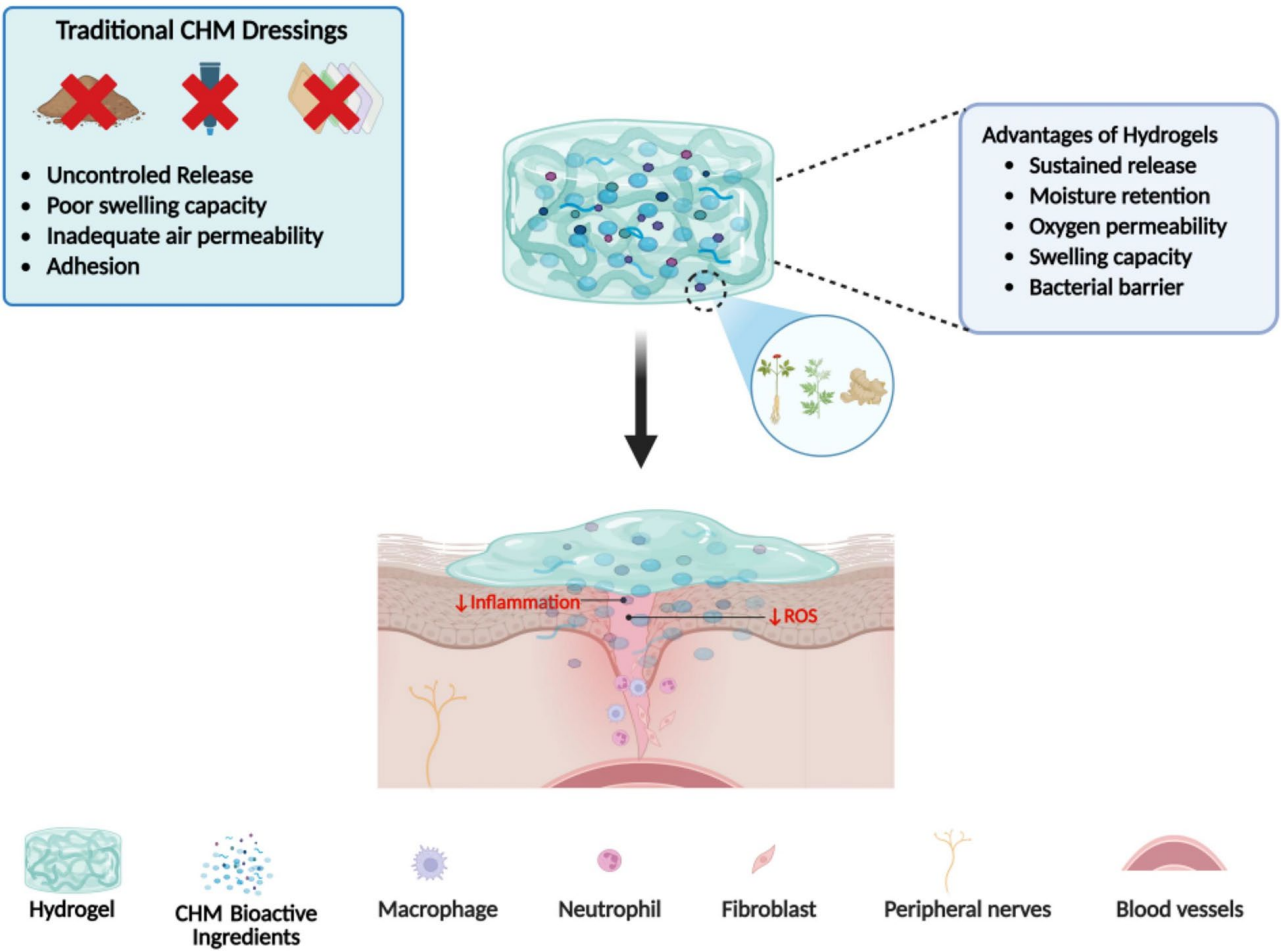
Schematic illustration of the structure and advantages of CHM-loaded hydrogels. Addressing the limitations of traditional CHM dressings (such as uncontrolled release, poor swelling capacity, poor permeability, and strong adhesion), CHM-loaded hydrogels offer sustained drug release, moisture retention, adequate oxygen permeability, swelling capacity, and bacterial barrier function

Hydrogels are three-dimensional cross-linked polymer networks formed from natural (e.g., gelatin, hyaluronic acid, chitosan, collagen, alginate) or synthetic (e.g., polyoxyethylene, polyethylene glycol, polylactic acid, polyacrylamide) hydrophilic monomers (Fig. 2). They offer excellent biocompatibility, moisture retention, oxygen permeability, and swelling properties [169]. The porous structure of hydrogels acts as both a barrier against bacterial invasion and a reservoir for the sustained release of CHM ingredients, protecting sensitive components from degradation [20, 21, 170]. Many hydrogel materials are Food and Drug Administration (FDA)-approved with well-established safety profiles. These advantages make CHM-loaded hydrogels an innovative platform for diabetic wound healing.

### CHM-loaded hydrogels

A range of CHM-loaded hydrogels has demonstrated pronounced efficacy in preclinical diabetic wound models. From a materials perspective, each hydrogel matrix offers distinct advantages, complementing the multi-target benefits of CHM. Gelatine methacryloyl (GelMA)-based hydrogels are photo-crosslinkable gelatin derivatives that enable in situ gelation and programmable drug release. Panax notoginseng saponins (PNS)-loaded GelMA/alginate hydrogels (GelMA/PNS/Alg@ IGF-1) enable sustained PNS release (88.2% by day 14) and significantly accelerate wound closure in diabetic rats [20].

Hyaluronic acid (HA)-based hydrogels are non-immunogenic glycosaminoglycans that promote angiogenesis and tissue remodeling through CD44 receptor-mediated pathway [171, 172]. When loaded with quercetin and oleic acid, HA hydrogels not only enhance wound healing but also reduce infection, with no reported adverse effects in a clinical study. Furthermore, the addition of quercetin effectively promoted the proliferation of keratinocytes compared to hyaluronic acid hydrogel alone [173]. Additionally, HA hydrogels loaded with Salvianolic acid B (SAB) improve mechanical strength, synchronize degradation with wound healing, and promote tissue repair [174, 175]. Mechanistically, hydrogen bonding between phenolic hydroxyl groups of SAB and the HA hydrazide groups enhances antioxidant activity and prevents SAB deactivation.

Chitosan-based hydrogels and derivatives significantly enhance stability and improve drug delivery [176–178]. Puerarin-loaded chitosan hydrogels (chitosan@puerarin) exhibit prolonged drug release (< 40% released by day 5) and significantly promoted fibroblast proliferation [179, 180]. Moreover, CHM ingredients with phenolic hydroxyl groups—such as baicalin, baicalein, or Ganoderma lucidum polysaccharide (GLP)—form dynamic imine bonds with carboxymethyl chitosan (CMCS), imparting self-healing properties to the hydrogels [141, 181].

Composite hydrogels, integrating multiple matrices, better meet the biological and mechanical demands of diabetic wound management. For example, poly(vinyl alcohol)–chitosan/sodium alginate (PCSA) loaded with curcumin offers robust mechanical properties, sustained drug release (12.5–25.9% after 12 h), and significantly promotes early angiogenesis in diabetic wound models [182]. The F127/F68 hybrid micelles loaded with phellopterin—a coumarin derived from *Angelica dahurica*—achieve sustained release of the poorly soluble compound, driving near-complete closure of 6-mm diabetic wounds within 10 days with a healing rate of 92.6%, which was significantly higher than the 70–80% observed in the traditional phellopterin and urea ointment group [183]. Collectively, CHM-loaded hydrogels offer a versatile platform for localized, sustained, and multifactorial therapy of diabetic wounds, significantly improving healing outcomes and reducing dressing change frequency.

### Stimuli-responsive CHM-loaded hydrogels

**Fig. 3:**
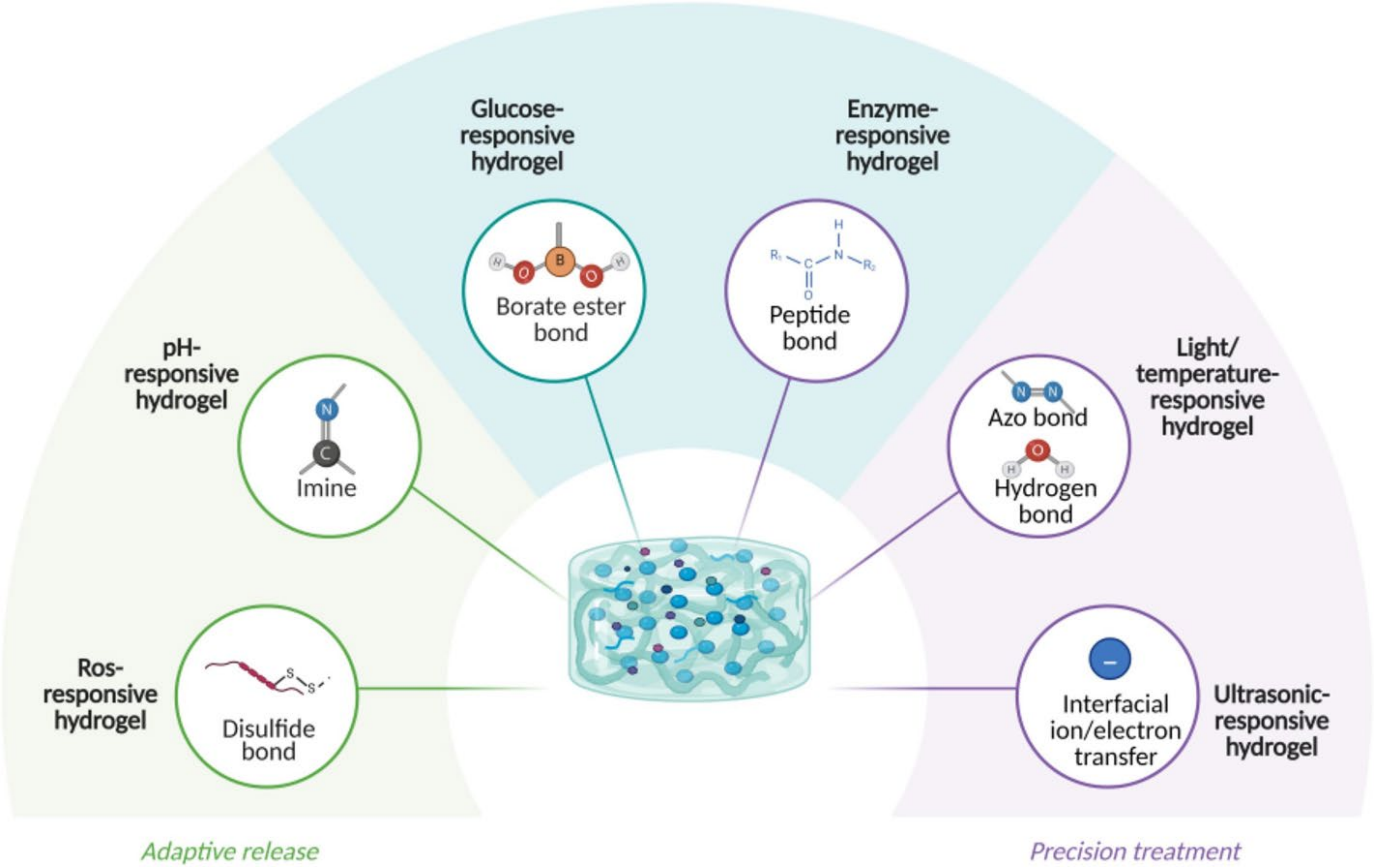
Schematic mechanistic illustration of stimuli-responsive CHM-loaded hydrogels for wound healing regulation. These hydrogels are designed to respond to specific pathological cues—including pH fluctuations, ROS, inflammatory mediators, hyperglycemia, and enzymatic activity—enabling precise, timely therapeutic interventions

In addition to conventional hydrogels platform, recent research has focused on developing intelligent, stimuli-responsive hydrogels that enable adaptive, on-demand drug release in response to the dynamic microenvironment of diabetic wounds [8, 25]. These advanced hydrogels are engineered to respond to specific pathological cues—including pH fluctuations, ROS, inflammatory mediators, hyperglycemia, and enzymatic activity—thereby enabling precise and timely therapeutic interventions (Fig. 3) [184–187].

ROS-responsive hydrogels incorporate redox-sensitive linkages that trigger drug release under oxidative stress, targeting specific areas of damage in diabetic wounds. For example, berberine-loaded HA-ferroelectric-β-cyclodextrin hydrogels demonstrated accelerated drug release (65% within 6 h) under high ROS conditions, nearly doubling the release efficiency compared to physiological conditions, thus improving therapeutic precision in diabetic wounds [188]. pH- and glucose-responsive hydrogels, typically constructed from ionizable polymers or boronic acid groups, dissociate under acidic or hyperglycemic conditions, triggering rapid drug release specifically in diabetic wounds. Dual-responsive hydrogels, such as those based on hydroxybutyl chitosan and 4-carboxyphenylboronic acid (CPBA), exhibit faster curcumin release under hyperglycemic and acidic conditions (51.3% release in high glucose vs 16.6% in normoglycemia), significantly improving infection control and wound healing [189]. Enzyme-responsive hydrogels use peptide linkers or natural polymers that degrade in the presence of wound-associated proteases, synchronizing drug release with local enzymatic activity to maximize therapeutic efficacy at the wound site [185].

Other responsive platforms, such as light-, temperature-, and ultrasound-responsive hydrogels, provide enhanced spatial and temporal precision [187, 190–192]. For example, curcumin-loaded conductive hydrogel can achieve on-demand release in response to ultrasound stimulation, enhancing neurogenesis, angiogenesis, and ECM remodeling in diabetic rat models [193]. Stimuli-responsive CHM hydrogels represent a new generation of wound dressings that align drug delivery with real-time changes in the wound microenvironment. This enhances therapeutic efficacy, minimizes off-target effects, and paves the way for personalized and advanced diabetic wound management.

## Innovations in CHM-derived exosome-like nanovesicles

**Fig. 4:**
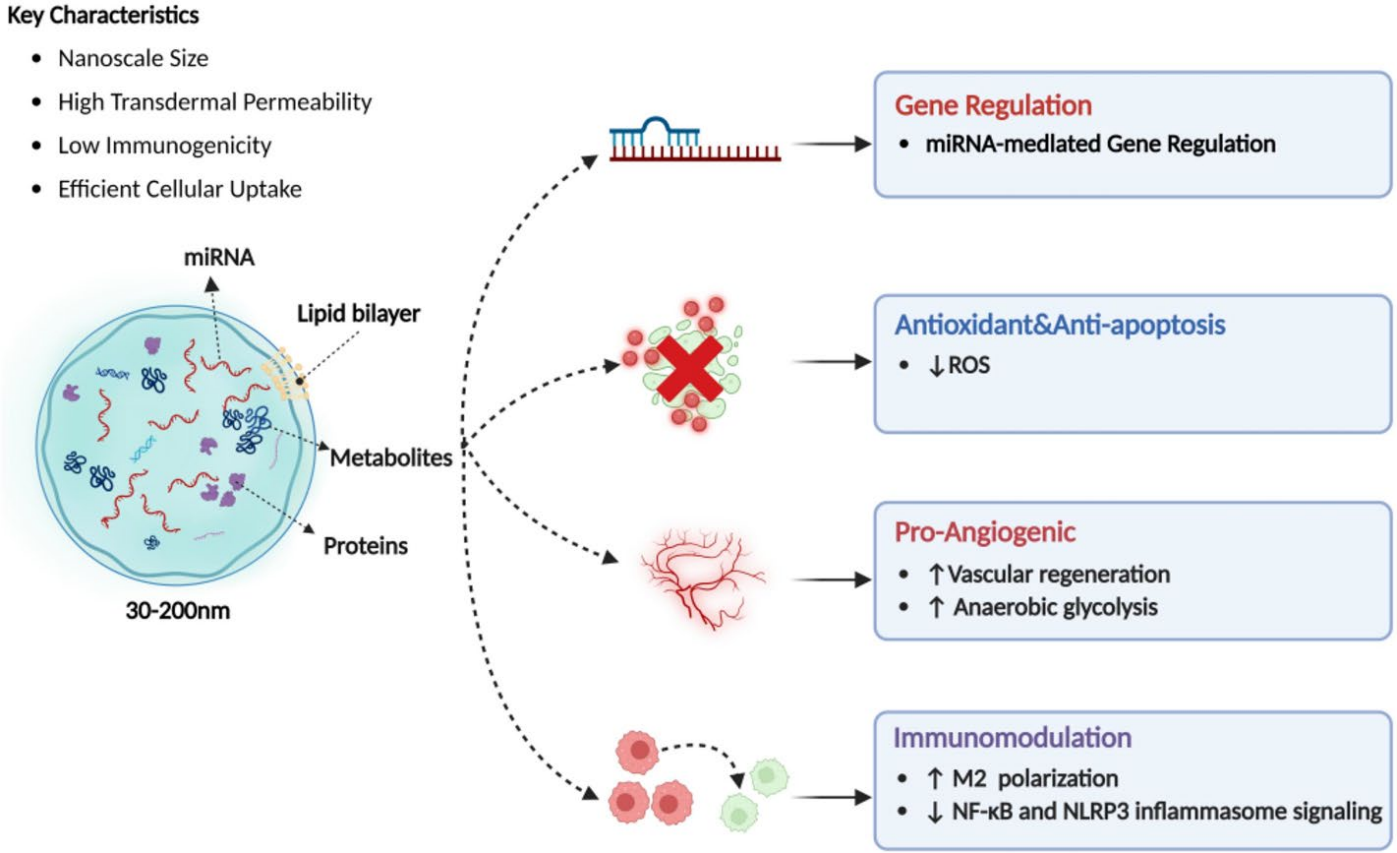
Schematic diagram of the multi-targeted regulatory mechanisms of CHM-derived exosome-like nanovesicles for diabetic wound healing. CHM-derived exosome-like nanovesicles are nano-sized extracellular vesicles (typically 30–200 nm in diameter) with lipid bilayers that enhance transdermal permeability, cellular uptake, and biocompatibility. These exosomes encapsulate miRNAs, proteins, and bioactive ingredients, exerting efficient therapeutic effects on diabetic wounds through miRNA-mediated gene regulation, antioxidant/anti-apoptosis, pro-angiogenesis, and immunomodulation

Exosomes are endogenously generated extracellular vesicles (EVs), typically ranging from 30 to 200 nm in diameter, which are secreted by nearly all eukaryotic cell types and characterized by a lipid bilayer membrane [194]. In plants, the biogenesis of exosomes remains under intense investigation. It is widely accepted that plant-derived EVs, including those from CHM, are generated through mechanisms such as the fusion of multivesicular bodies (MVBs), vacuoles, or exocyst-positive organelles (EXPO) with the plasma membrane [195]. The phospholipid bilayer structure of these exosomes is structurally similar to that of the mammalian cell membrane, conferring important biological advantages, including superior biocompatibility and low immunogenicity in vivo [196]. Moreover, their inherent nanoscale size enhances transdermal permeability, facilitates cellular uptake, and improves the overall bioavailability compared to conventional herbal decoctions (Fig. 4) [197].

Exosomes serve as natural nanocarriers that encapsulate a variety of cargos, including proteins, lipids, and non-coding RNAs such as miRNAs and messenger RNAs (mRNAs). Notably, exosomal miRNAs are potent post-transcriptional gene regulators, mediating intercellular and cross-kingdom communication, which positions them as key effectors in therapeutic applications [198]. PELNs also efficiently load and deliver various bioactive ingredients from CHM, exhibiting similar pharmacological effects as the original herbal compounds [199].

Recent advances have highlighted the therapeutic advantages of PELNs in diabetic wound healing. For example, ginseng-derived exosomes (GExos), naturally enriched with active ginsenosides (e.g., Rg1, Re, Rb1) and functional miRNAs, promote angiogenesis by enhancing anaerobic glycolysis, reducing oxidative stress, and supporting endothelial tube formation, even under hyperglycemic conditions [197]. GExos are rapidly internalized by endothelial cells via lipid fusion or clathrin-mediated endocytosis, with uptake rates approaching 39.8% within 3 h, enabling rapid therapeutic impacts [198]. Similarly, turmeric-derived exosomes deliver both plant ingredients (e.g., choline, polygodial, ar-turmerone, retinene) and miRNAs, promoting wound healing by activating the Nrf2/HO-1 antioxidant pathway, suppressing pro-apoptotic signals (e.g., regulating the BAX/Bcl-2 ratio) and facilitating M2 macrophage polarization via the TLR4–MyD88 signaling axis [200]. Exosomes derived from *Houttuynia cordata* Thunb. and *Portulaca oleracea* L. have also shown similar therapeutic benefits in diabetic wound repair, likely through parallel mechanisms such as reducing inflammation and promoting tissue re-epithelialization [201, 202]. Furthermore, plant-derived exosome-like nanovesicles isolated from *Rhodiola rosea* and *Taraxacum officinale* exhibit significantly superior anti-inflammatory and anti-fibrotic efficacy compared to their respective traditional aqueous extracts [203]. This underscores that the natural phospholipid bilayer of PELNs efficiently protects active cargo from enzymatic degradation and preserves critical bioactivity that is otherwise compromised during conventional decoction processing [204, 205].

Although research on PELNs is in its early stages, several clinical trials have demonstrated preliminary safety and therapeutic potential in conditions such as oral mucosal inflammation (NCT01668849), inflammatory bowel disease (NCT04879810), and cardiovascular disease risk (NCT04698447). These findings suggest that PELNs not only exhibit anti-inflammatory and antioxidant properties but also show significant promise for wound healing and tissue regeneration. However, large-scale, randomized clinical trials (RCTs) are urgently warranted to validate their safety, optimal dosing, and therapeutic effectiveness in human patients with diabetic wounds in the future.

In conclusion, PELNs represent a new generation of natural nanocarriers that provide efficient transdermal delivery and sustained therapeutic action of herbal bioactives and vesicle-encapsulated miRNAs/proteins. With low immunogenicity, excellent biocompatibility, and minimal cytotoxicity, PELNs are emerging as a highly promising strategy to overcome the limitations of conventional diabetic wound therapies [24, 196].

While this review primarily focuses on CHM-loaded hydrogels and PELNs, other advanced delivery strategies—including microneedle arrays, electrospun nanofibers, and lipid-based nanoparticles—are also emerging as potent tools for diabetic wound management [206–208]. For instance, microneedles can painlessly bypass the hyperkeratotic tissue common in diabetic ulcers, facilitating the targeted transdermal delivery of CHM extracts into the deeper dermis [209]. Electrospun nanofibers provide biomimetic scaffolds with highly porous structures that optimize oxygen exchange and exudate management, both of which are severely compromised in diabetic microenvironments [210]. Furthermore, lipid-based nanoparticles can efficiently encapsulate poorly water-soluble CHM monocomponents, protecting sensitive antioxidants from rapid enzymatic degradation in the protease-rich wound milieu [211]. Although the integration of these alternative platforms with CHM agents is still evolving, they represent a highly promising frontier for expanding personalized therapeutic options in diabetic wound care.

## Conclusion and perspective

Diabetic wounds represent one of the most intractable complications of diabetes, characterized by a complex, self-amplifying pathological microenvironment involving metabolic imbalance, chronic inflammation, oxidative stress, impaired angiogenesis, neuropathy, immune dysfunction, recurrent infection, and dysfunction of resident cells [9, 14, 55, 75]. Conventional clinical management—including debridement, anti-infection strategies, and pharmacotherapy—remains inadequate to break this vicious cycle and restore effective healing. By contrast, CHM has demonstrated unique advantages owing to its rich repertoire of bioactive ingredients. Many CHM ingredients even exhibit pleiotropic activities via multi-target and multi-pathway regulation in diabetic wound management, simultaneously attenuating inflammation, scavenging ROS, promoting angiogenesis, facilitating neuroprotection, modulating immune responses, supporting ECM remodeling, and suppressing infection, thus enabling integrated and comprehensive therapeutic outcomes.

Recent advances in biomaterials and nanotechnology have further opened new avenues for CHM-based therapies. Innovative delivery platforms, such as CHM-loaded hydrogels and PELNs, significantly enhance the bioavailability, targeting precision, and therapeutic efficacy of CHM [123, 124].

Hydrogels provide on-demand, microenvironment-responsive release, acting as both physical barriers and biological stimulants to accelerate repair. Early clinical trials of PELNs further show promise for safety and efficacy in modulating inflammation and ROS in different diseases. We emphasize that large-scale, multicenter randomized controlled trials directly comparing identical CHM formulations delivered via hydrogels or PELNs versus traditional TCM decoctions or plasters are urgently warranted. The current literature remains predominantly in the proof-of-concept stage, with most studies evaluating innovative platforms against untreated or vehicle controls rather than against traditional counterparts. Rigorous comparative effectiveness trials are essential to validate the mechanistic superiority suggested by independent pharmacokinetic studies.

To fully realize the therapeutic potential of CHM in diabetic wound management, more research are needed. The complex pharmacology of CHM warrants further systematic exploration using advanced technologies, such as transcriptomics, proteomics, metabolomics, and single-cell sequencing, which would clarify network-level mechanisms of action, identify novel molecular targets, and guide function-oriented ingredient discovery and rational formulation design. The design and optimization of more next-generation biomaterials—such as intelligent hydrogels sensitive to pH, ROS, or enzymatic activity—may enable precision, on-demand delivery of CHM actives, maximizing local efficacy and minimizing systemic side effects. Most importantly, high-quality, multicenter randomized controlled trials (RCTs) are urgently needed to provide robust evidence for the efficacy, safety, and optimal use of CHM-based wound therapies. As the clinical and commercial prospects for CHM-based therapies expand, rigorous standardization of raw materials, extraction processes, ingredient quantification, and formulation is also essential.

In summary, CHM and its advanced delivery platforms represent a promising multi-target approach for overcoming the clinical and mechanistic barriers in diabetic wound healing. By integrating traditional herbal wisdom with cutting-edge biomaterial and nanotechnological innovation, these strategies lay a robust foundation for future evidence-based therapies. Effective translation of CHM research from bench to bedside will require continued collaboration among herbal pharmacologists, materials scientists, clinicians, and regulatory agencies.

## Data Availability

No datasets were generated or analysed during the current study.

## Abbreviations

AGEs: Advanced glycation end products
AKT1: RAC-alpha serine/threonine-protein kinase
Ang-1: Angiopoietin-1
APS: Astragalus polysaccharide
ASA VI: Asperosaponin VI
AS-IV: Astragaloside IV
BBR: Berberine
BDNF: Brain-derived neurotrophic factor
CASP3: Caspase-3
Ccl2: CC-chemokine ligand 2 (chemokine C-C motif ligand 2)
CD44: Cluster of differentiation 44
CHM: Chinese herbal medicine
CMCS: Carboxymethyl chitosan
CGRP: Calcitonin gene-related peptide
CPBA: 4-Carboxyphenylboronic acid
DPN: Diabetic peripheral neuropathy
ECM: Extracellular matrix
EndMT: Endothelial-to-mesenchymal transition
eNOS: Endothelial nitric oxide synthase
EPCs: Endothelial progenitor cells
ER: Endoplasmic reticulum
EVs: Extracellular vesicles
EXPO: Exocyst-positive organelles
FDA: Food and Drug Administration
FGF: Fibroblast growth factor
FGF2: Fibroblast growth factor 2
GAP-43: Growth-associated protein 43
GDNF: Glial cell line-derived neurotrophic factor
GelMA: Gelatine methacryloyl
GExos: Ginseng-derived exosomes
GLP: Ganoderma lucidum polysaccharide
GPx: Glutathione peroxidase
GSH: Reduced glutathione
HA: Hyaluronic acid
HGF: Hepatocyte growth factor
HIF-1α: Hypoxia-inducible factor-1α
HO-1: Heme oxygenase-1
IDF: International Diabetes Federation
IGF-1: Insulin-like growth factor 1
IL-1β: Interleukin-1β
IL-6: Interleukin-6
IL-10: Interleukin-10
IL-13: Interleukin-13
IL-17: Interleukin-17
IRAK1: Interleukin-1 Receptor-Associated Kinase 1
IRF-5: Interferon regulatory factor 5
IRF8: Interferon regulatory factor 8
KLF4: Krüppel-like factor 4
lncRNA GAS5: Long noncoding RNA growth arrest-specific 5
MBP: Myelin basic protein
MMP: Matrix metalloproteinase
MMP9: Matrix metalloproteinase 9
MMPs: Matrix metalloproteinases
MnSOD: Manganese superoxide dismutase
mRNAs: Messenger RNAs
MVBs: Multivesicular bodies
MyD88: Myeloid differentiation primary response 88
NADPH: Nicotinamide adenine dinucleotide phosphate
NETs: Neutrophil extracellular traps
NF-κB: Nuclear factor-κB
NGF: Nerve growth factor
NLRP3: NOD-, LRR- and pyrin domain-containing protein 3
NO: Nitric oxide
NPWT: Negative pressure wound therapy
OXPHOS: Oxidative phosphorylation
PAD4: Peptidylarginine deiminase 4
PAK2: P21-activated kinase 2
PCSA: Poly(vinyl alcohol)–chitosan/sodium alginate
PDGF: Platelet-derived growth factor
PECAM-1: Platelet/endothelial cell adhesion molecule
PELNs: Plant-derived exosome-like nanovesicles
PF: Paeoniflorin
PGC-1α: Peroxisome proliferator-activated receptor-γ coactivator 1α
PKC: Protein kinase C
PNS: Panax notoginseng saponins
QS: Quorum sensing
RAGE: Receptor for AGEs
RCTs: Randomized controlled trials
RJP: Ruyi Jinhuang Powder
ROS: Reactive oxygen species
RR: Rehmanniae Radix
SAB: Salvianolic acid B
SDF-1α: Stromal Cell-Derived Factor 1 alpha
SHXY: Sanhuang Xiaoyan Formula
SOD: Superoxide dismutase
STAT1: Signal transducer and activator of transcription 1
STAT6: Signal transducer and activator of transcription 6
T-AOC: Total antioxidant capacity
TGF-β: Transforming growth factor-β
TGF-β1: Transforming growth factor-β1
Tie-2: Tyrosine kinase receptor 2
TIMP1/TIMP-1: Tissue inhibitor of metalloproteinases 1
TNF-α: Tumor necrosis factor-α
TRAF6: TNF Receptor-Associated Factor 6
TSP2: Thrombospondin 2
TYO: Tangzu Yuyang Ointment
USP7: Ubiquitin-specific protease 7
VEGF: Vascular endothelial growth factor
YAP: Yes-associated protein
Ym1: Chitinase-like protein 3 (Chi3l3)
